# Draft Genome Sequence of *Alternaria alternata* ATCC 34957

**DOI:** 10.1128/genomeA.01554-15

**Published:** 2016-01-14

**Authors:** Hai D. T. Nguyen, Christopher T. Lewis, C. André Lévesque, Tom Gräfenhan

**Affiliations:** a Ottawa Research and Development Centre, Agriculture and Agri-Food Canada, Ottawa, Ontario, Canada; b Grain Research Laboratory, Canadian Grain Commission, Winnipeg, Manitoba, Canada

## Abstract

We report the draft genome sequence of *Alternaria alternata* ATCC 34957. This strain was previously reported to produce alternariol and alternariol monomethyl ether on weathered grain sorghum. The genome was sequenced with PacBio technology and assembled into 27 scaffolds with a total genome size of 33.5 Mb.

## GENOME ANNOUNCEMENT

The genus *Alternaria* was originally described based on *A. tenuis* (1). Almost 100 years later, *A. tenuis* and *Torula alternata* (2) were synonymized with *A. alternata* (3). Then, another century later, the genus *Alternaria* was drastically redefined based on multilocus phylogenetic analyses (4). Currently, the species *A. alternata* comprises 35 morphospecies, which cannot be distinguished phylogenetically (5). *A. alternata* has no known sexual stage and produces characteristic dark-colored phaeodictyospores in chains with a beak of tapering apical cells. This species is found to infect hundreds of host species of plants, causing leaf spots and other diseases. A wide range of metabolites have been reported for *A. alternata* (6, 7), including alternariol (AOH), alternariol monomethyl ether (AME), altenuene (ALT), altertoxin I, and tenuazonic acid (TEA) in weathered grain sorghum by isolate RL-8442-2 (ATCC 34957) (6, 7). For over 40 years, this isolate has retained its propensity to form these “emerging” mycotoxins in culture (K. Sivagnanam, personal communications). Here, we report the whole-genome sequence of this strain of *A. alternata*, ATCC 34957. This information will be used to discern the synteny of genes involved in the biosynthesis pathways of secondary metabolites.

Genomic DNA was isolated using a large-scale version of a previously described method with an additional 5M/3M potassium acetate/acetic acid step and RNase treatment (8). Extracted DNA was sheared using the HydroShear Plus DNA shearing instrument (Digilab, Marlborough, MA, USA) and then size-selected on a BluePippin system (Sage Science Inc., Beverly, MA, USA). The DNA libraries were prepared following the Pacific Biosciences 20-kb Template Preparation Using BluePippin Size-Selection System protocol. The DNA damage repair, end repair, and SMRT bell ligation steps were performed as described in the template preparation protocol with the SMRTbell template prep kit version 1.0 reagents (Pacific Biosciences, Menlo Park, CA, USA). The libraries were sequenced on a PacBio RSII instrument using the MagBead OneCellPerWell loading protocol, DNA sequencing kit 2.0, SMRT cells version 3, and 3-h movies. *De novo* genome assembly was performed with Celera WGS assembler version 8.2, using the PBcR pipeline for high-noise single-molecule sequencing data (9). Genome statistics were determined with QUAST version 2.3 (10). CEGMA version 2.5 (11) was run on the scaffolds to detect the percentage of conserved eukaryotic genes.

*A. alternata* has 9 to 11 chromosomes and a genome of up to 33.6 Mb (12). Our *A. alternata* ATCC 34957 genome is contained in 27 scaffolds spanning 33.5 Mb with 16× coverage. The largest scaffold is 3.97 Mb and the *N*_50_ is 2.83 Mb. Scores of 96% and 97% were obtained from the complete and partial gene sets, respectively, using CEGMA. This is a draft genome but close to being complete because the telomeric sequence motif (13, 14) was found at both ends of four scaffolds (scaffolds 8, 9, 10, and 11) and at one end of seven scaffolds (scaffolds 2, 3, 4, 5, 7, 12, and 13).

### Nucleotide sequence accession numbers.

This whole-genome shotgun project has been deposited at DDBJ/EMBL/GenBank under accession number LMXP00000000. The version described in this paper is the first version, LMXP01000000.
